# Chimeric antigen receptor-engineered T cells for cancer immunotherapy: progress and challenges

**DOI:** 10.1186/1756-8722-6-47

**Published:** 2013-07-08

**Authors:** Ethan Q Han, Xiu-ling Li, Chun-rong Wang, Tian-fang Li, Shuang-yin Han

**Affiliations:** 1 Drexel University College of Medicine, Philadelphia, PA 19129, USA; 2 Translational Research Center, Zhengzhou University People’s Hospital, Zhengzhou, Henan 450003, China; 3 Rush University Medical Center, Chicago, IL 60612, USA

**Keywords:** Chimeric antigen receptor, Adoptive immunotherapy, Single chain variable fragment, T cell receptor

## Abstract

Recent years have witnessed much progress in both basic research and clinical trials regarding cancer immunotherapy with chimeric antigen receptor (CAR)-engineered T cells. The unique structure of CAR endows T cell tumor specific cytotoxicity and resistance to immunosuppressive microenvironment in cancers, which helps patients to better tackle the issue of immunological tolerance. Adoptive immunotherapy (AIT) using this supernatural T cell have gained momentum after decades of intense debates because of the promising results obtained from preclinical models and clinical trials. However, it is very important for us to evaluate thoroughly the challenges/obstacles before widespread clinical application, which clearly warrants more studies to improve our understanding of the mechanism underlying AIT. In this review, we focus on the critical issues related to the clinical outcomes of CAR-based adoptive immunotherapy and discuss the rationales to refine this new cancer therapeutic modality.

## Introduction

Adoptive immunotherapy (AIT) using chimeric antigen receptor-engineered T cells is a promising approach in cancer treatment. Significant progresses made in the past decades have contributed to the development of more efficient antitumor immunotherapy. Incorporation of a single chain variable fragment (scFv) of a tumor antigen specific antibody and signaling domains of T cell receptor (TCR) renders chimeric antigen receptor (CAR) the specificity of antibody as well as the cytotoxicity of cytotoxic T lymphocytes (CTLs) [1]. CAR endows T cells antigen specific recognition, activation and proliferation in an MHC independent manner. In addition, CAR bypasses many mechanisms through which cancer cells escape immunorecognition. These mechanisms include down-regulation of the MHC, reduced expression of costimulatory molecules, induction of suppressive cytokines and recruitment of regulatory T cells. Besides these beneficial effects, the technical feasibility of CAR makes it even more attractive in the development of cancer adoptive immunotherapy. The observations from preclinical and clinical studies have revealed a very encouraging therapeutic efficacy of the CAR-mediated immunotherapy in a variety of cancers including lymphoma, chronic lymphocytic leukemia, melanoma, and neuroblastoma [2]. Although there are many obstacles needed to be overcome before its widespread clinical use, it is still highly likely for the CAR-based immunotherapy to become a mainstay in clinical oncology field due to its improved tumoricidal activity. In this review, we will briefly discuss the critical issues related to the antitumor efficacy including the structure of CAR, the potential of T cells subsets, the gene modification of lymphocytes, the preconditioning regimens, etc.

### Promising results of clinical trials

The promising observations made from in vitro studies and animal models have greatly encouraged physicians/scientists to conduct clinical studies. The efficacy and safety of adoptive CAR-engineered T cells have been evaluated in multiple oncological settings. Currently, about 30 ongoing clinical trials are under evaluation (http://www.clinicaltrials.gov). Many advances have been reported regarding the outcomes of these early clinical studies (Phase I/II) including the effect on hematological malignancies and some solid tumors.

To date, the most encouraging clinical observations have been achieved from patients with chronic lymphocytic leukemia (CLL) and lymphoma treated by CD19-orientated CAR T cells [3]. CD19 is expressed on most types of B-cell leukemia/lymphoma but absent in non-B lineage cells. Dr. June and the colleagues have made significant contributions that have greatly improved our understanding of the CAR T cells. In one of their reports, 2 out of 3 refractory CLL patients receiving CD19 CAR T cells therapy achieved complete response (CR), and another patient demonstrated partial response (PR) [4]. This proof of concept clinical investigations have demonstrated an excellent cell engraftment (up to 3 log expansion) and tumor cell lysis, eventually leading to complete remission. This pioneer work inspired numerous clinical studies focusing on CD19 for evaluating CAR technology. CD19 CAR studies account for one third of ongoing clinical trials with constantly increasing enrollments. Recent reports from seven academic institutes showed 25% overall CR rate (for review see [3,5,6]). In addition to CD19, other molecules such as CD20, CD22, CD30 and CD33 have also been tested for their potential as therapeutic targets for hematological malignancies [7,8].

In parallel with the clinical trials in hematological malignancies, CAR-based therapy has also been conducted in solid tumors. CAR T cells targeting diasialoganglioside (GD2) antigen and L1-cell adhesion molecule (L1CAM) were generated for treatment of neuroblastoma [9]. Dr. Louis and the colleagues generated the first-generation CAR and modified the EBV-specific T cells. Enhanced in vivo persistence and survival of the adoptively transferred CAR-modified T cells resulted in a durable and complete response in 3 out of 11 patients [10]. Dr. Lamers’ team generated the CAR targeting carboxyl anhydrase IX (CAIX) to treat metastatic renal cell carcinoma and there was no objective clinical responses observed in any of the treated patients [11]. In addition, CARs have been generated against a number of surface molecules on solid tumors, including HER2 for colorectal cancer [12], folate receptor-α for ovarian cancer [13], carcinoembryonic antigen (CEA) for colorectal and breast cancer [14], epidermal growth factor receptor variant III (EGFRvIII) for neuroblastoma [15], and prostate-specific membrane antigen (PMSA) for prostate cancer [16]. Targeting solid tumors is more challenging compared to the treatment of hematological malignancies because of rare target antigens, poor T cells trafficking to tumor site, less cytotoxicity in local tumor immunosuppressive microenvironment.

Despite small sizes and different protocols, these phase I/II studies did demonstrate the feasibility and safety of CAR-based immunotherapy for the treatment of some types of cancer. While adverse effects are generally tolerable, the clinical efficacy of the first generation CAR is often insufficient. The CARs of the second and third generations have demonstrated improved efficacy and durable response, which motivated scientists for dynamic and innovative research in this field.

### Evolving architecture of CAR

CARs have evolved considerably over the past decades. Basically, CAR is comprised of an extracellular antigen-binding moiety, a hinge and transmembrane (TM) domain, and an intracellular signaling element. The initial CAR of the first generation provides a proof of concept of the targeting and activation of T cells. The CAR of the second and third generations have been developed by addition of dual or triple costimulatory signaling domains in order to increase their cytotoxicity [17]. The most important function of multiple signaling receptors is to enhance signaling strength and persistence, subsequently increasing in their overall potency.

Targeting, the first step for the redirected T lymphocytes to function, is initiated at the single chain fragment variable (scFv) region of extracellular domain. The scFv determines the specificity of CAR and binds to target molecules through a similar mechanism as antigen-antibody interaction. Many factors including the affinity of scFv, the spatial topologic structure of epitopes, and antigen expression levels on tumor cells, have to be considered to optimize the CAR-mediated T-cell response [18]. Besides widely used scFv in many CARs, ligand or receptor can also be used as targeting moiety because the corresponding receptor or ligand are often overexpressed on tumor cells, such as peptide against vascular endothelial growth factor receptor (VEGFR) [19], and receptor for NKG2D [20]. The potential immunogenicity of murine-derived scFv limits the use of CAR in clinical setting. Utilizing humanized or fully human antibodies to construct CAR may lower the immunogenicity and avoid immune-mediated recognition leading to elimination of the genetically modified T cells.

A flexible hinge region between the targeting moiety and the TM domain seems to be important for accessibility to the epitope of the CAR-grafted T cells [21]. A study performed by Dr. Guest demonstrated that a flexible hinge region is needed for targeting membrane-proximal epitope, instead of membrane-distal epitopes [22]. Considering the spatial structure of the antigen, efficient antigen recognition of CAR might be modulated by different choices of target epitopes. Also, the TM domain is a pivotal region for CAR function, more specifically, the oligomeric status of CARs. Dr. Bridgeman and colleagues have demonstrated that CAR containing a CD3*ζ* TM domain form homodimers and are incorporated into the endogenous T cell receptor (TCR) complex [23]. Various transmembrane regions have been employed in CAR including those derived from CD28, CD3z, CD8, CD4, FcRγ, etc.

Intracellular signaling domain is of vital importance for CAR T cells to fulfill their antitumor function. Therefore, new generation of CAR contain a second signaling domain by adding the cytoplasmic domain of costimulatory receptors such as CD28, CD137 (4-1BB), CD134 (OX40), or inducible costimulator (ICOS). Both in vitro and in vivo studies have demonstrated that, in response to the target antigen, the modified T cells with the second or third generation CARs usually demonstrate sustained proliferation, enhanced production of cytokines and tumor lytic activity, and reduced activation-induced cell death. In a study of mouse model for comparison of the first and second generation CARs, Dr. Savoldo demonstrated the greater persistence of T cells expressing a CD28/CD3ζ-based CAR compared with T cells transduced by CAR with CD3ζ signaling only [24]. The third generation CAR usually shows better attributes of T cells than second generation due to a synergistic effect of triple signaling domains [25]. Nowadays, most researchers prefer to use the second generation CAR because of the concern about possible signaling leakage of the third generation CAR due to reduced activation threshold. As for costimulatory molecules per se, we cannot reach a clear conclusion about which one is better than the other. Previous observations indicate that inclusion of CD28 significantly increases IL-2 production compared to the inclusion of other molecules. The inclusion of CD137 may improve survival, and the use of the ICOS signaling seems the most efficient in target cell lysis [26-28].

Collectively, optimal designs of the CAR and a careful choice of the tumor associate antigen (TAA) are indispensable for the CAR-mediated therapy to attain a significant response. Although CARs in many clinical trials greatly differ in their configurations, comprehensive comparisons are still lacking. With the clinical results in mind, we need to attain a better understanding of optimal CAR signaling to promote sustained T-cell function and survival, to avoid undue proliferation, and to prevent premature death and rapid exhaustion. It is also desirable to add more elements such as homing and suicide genes to improve their functionality. It is imperative to develop a more competent and safer architecture of CAR, so-called the fourth generation CAR. Optimized design of the new generation CAR may be achieved by more extensive basic studies investigating the spatiotemporal dynamics of CAR-mediated molecular events and unraveling the molecular basis of T-cell activation by CARs.

### Therapeutic potential of T cell subsets

In the past decades, researchers have tried to generate sufficient quantity of antigen-specific T cells based on the notion that the absolute number of transferred T cells is correlated with tumor responses. Most clinical trials take use of mixed populations of CD3^+^ T cells containing antigen-specific CD8^+^ and CD4^+^ T cells, and the phenotypic and functional attributes of the transferred T cells are subject to natural variation. As the heterogeneity and potential variable of polyclonal unselected cells could affect the efficacy and safety of cancer immunotherapy, precise detection and careful selection of the potentially most potent T cell subsets would increase their adoptive transfer efficiency.

It becomes increasingly clear that adoptive transfer of the less-differentiated T cell subsets is associated with superior T cell engraftment, persistence, and antitumor immunity, thus highly correlating with objective clinical responses [29]. An in vivo study demonstrated that adoptive transfer of central memory T cells (T_CM_) induced complete responses at the tested cell dose, whereas the mice in group of receiving effector memory T cells (T_EM_) suffered uncontrolled tumor growth [30]. This difference can be deciphered by the fact that T_CM_ cells can self-renew and differentiate into effector T cells in vivo, while T_EM_ cells have lost this plasticity. Subsequently, a subset of stem cell memory T cell (T_SCM_) has been demonstrated to have superior ability to mediate cancer regression compared to the T_CM_ and T_EM_ populations [31]. Therefore, ex vivo sorting/selection for superior T cell subsets like T_CM_ or T_SCM_ cells prior to adoptive transfer and/or gene transfer could improve in vivo persistence and therapeutic efficacy. Clinical trials have been conducting to evaluate the potential of the T cell subsets from the peripheral blood [32].

Many strategies have been utilized to obtain sufficient numbers of defined therapeutically clinical-grade CTL effectors. Current protocol includes isolation and activation using agonistic antibodies CD3 or *γ*-irradiated allogeneic peripheral blood mononuclear cell (PBMC) vs EBV-transformed B lymphoblastoid cell line (LCL) feeders, and a subsequent rapid expansion of cells in the presence of IL-2. Since ex vivo expansion inevitably drives T cell differentiation and results in the loss of in vivo antitumor efficacy, new strategies for handling cultured T cells have been developing recently. Traditional method for cell processing has been replaced by the automated manufacturing system. Activation of T cells can be induced by various kinds of antigen-non-specific artificial APCs (aAPCs) such as magnetic CD3/CD28 beads and K32 cell line (CD32-transfected precursor K562 chronic erythroleukemic cell line) [33].

IL-2 has been used for long time for T cell expansion. Recent evidence suggests that IL-2 may have some negative effects by depleting memory T cells and increasing the number of tumor protecting regulatory T cells (Tregs) [34]. In contrast, IL-15 can increase the persistence of CD8^+^ memory T lymphocytes, prevent Fas induced apoptosis and overcome tolerance of tumor specific CD8^+^ T cells [35]. The expansion of naive CD8^+^ T cells in the presence of IL-15 generates cells with the phenotypic and functional properties of naturally occurring T_CM_ with superior antitumor capability. Therapeutic potentials of small molecular modulators in critical metabolic and developmental pathways involved in T cell differentiation have also been evaluated including those in the PI3K-AKT-mTOR and Wnt-β-catenin pathways [36].

Our understanding of the fundamental properties of heterogeneous subsets of T cells keep improving and mounting evidence indicates that selection of defined populations for gene modification is necessary to maximize the outcome of adoptive immunotherapy for cancer. The phenotypic and functional diversity of the genetically modified T cells may provide opportunities to enhance cancer immunotherapy. Because of severe adverse events reported in CAR clinical trial, it may become requisite to choose the defined T cell subsets in context of clinical setting. With recent advent of cell bioprocessing and genetic engineering technology, it is now possible to generate tumor-reactive CD8^+^ T cells of a defined subset. The in-depth understanding of phenotypes and functions of T cell subsets will have profound impact on future CAR T cell therapy.

### Improved methods of T cell gene modification

CAR-based immunotherapy is dependent on the efficient genetic modifications of the infused T cells. Since extensive ex vivo T cell expansion is prerequisite for adoptive cell transfer, genetic modifications must be stable and require vector integration. For this reason, transduction using chromosome-integrating vectors like retroviruses or lentiviruses is superior to transfection with transient gene expression of DNA in genetically modified T cell therapy.

To date, most studies of CAR-related therapy utilize retroviruses or lentiviruses for gene delivery. Data from several sources indicate that retroviral vectors do not elicit clinically significant genotoxicity when used to deliver therapeutic genes to T cells [37]. Dr. Scholler reported long-term results from three clinical trials to evaluate retroviral vector*–*engineered T cells. In a clinical analysis of HIV and malignancy, which included over 200 patients with the follow-ups for as long as 11 years, transduced T cells were detected in 98% of patients with no evidence of integration-induced immortalization [38]. Lentivirus vectors have some advantages over retroviral vectors as they can transduce non-dividing cells. In addition, lentiviral vectors have higher cargo capacity and reduced susceptibility to gene silencing. Because of its safety profile, it is expected that the future use of lentiviral vectors will keep increasing [39]. Besides, Ad5/F35 adenoviral vector transduction were reported to mediate gene transfer in up to 10% of resting T cells and 30–45% of T cells after activation with phyto-haemagglutinin. Ad5/F35 vectors hold promise for adoptive transfer of engineered lymphocytes in clinical situations where expression of a transgene for less than a week is required [40].

Along with the advance of viral vectors, non-viral gene transduction systems have been developed for clinical trials as well. The lower costs and better safety profile of non-viral vectors have attracted much attention. Transposon-based system is one of the promising methods because of much higher efficiency of the transgene integration. Sleeping Beauty was the first transposon system used for modification of human T lymphocytes and demonstrated stable expression of CAR with killing of targeted cancer cells in vitro as well as in animal models [41]. PiggyBac transposons mediate stable gene expression in about 20% of primary T cells before selection, and the rate was increased to 40% after selection. The expression can sustain for over 9 weeks in culture through multiple logs of expansion [42]. Thus far, both Sleeping Beauty and PiggyBac have been used for gene modification of human T cells with CAR for HER2, CD19 and so on. RNA-based electroporation of lymphocytes is also an attractive approach because of the almost 100% transduction efficiencies, less genotoxic and transient expression [43]. The clinical trial was planned to treat the hematological malignancies.

While genetic-modification strategies provide us with the opportunity to harness the immune system against malignant disease, several issues must be taken into consideration when selecting the technology used to engineer cellular immunity. These factors include the size, number, and expression level of the transgenes, the requirement for permanent or transient genetic modification, and different delivery approaches for either constitutive or inducible transgene expression.

### Optimizing regimen of host preconditioning

Numerous animal experiments as well as clinical studies in humans strongly suggest that the effectiveness of adoptively transferred T cells can be reinforced when combined with conventional cytotoxic agents such as cyclophosphamide and fludarabine, and occasionally concomitantly used with a low-dose irradiation. It was observed in adoptive transfer of tumor infiltrated lymphocytes (TILs) that preconditioning with systemic non-myeloablative chemotherapy can induce clear and reproducible responses in a substantial percentage (~50%) of patients [44]. Therefore, most recent clinical trials using CAR-engineered T cells incorporate a lymphodepletion step, which has demonstrated an increased antitumor response [45,46]. Clearly, preconditioning chemotherapy plays a critical role in the efficacy of targeted T-cell therapy.

Several mechanisms may contribute to the increased efficacy of T cell-based immunotherapy in the lymphopaenic environment. Deletion of the suppressive CD4^+^CD25^+^ regulatory T (T_Reg_) cells in preconditioning recipient will help the infused T cells to function normally [47]. Competition for the limited amount of the cytokines (so-called cytokine sink) between transferred and endogenous T cells interferes CD8^+^ T cell homeostasis. Preconditioning will eliminate cytokine sinks and creates space for the expansion of infused cells [48]. Transient eradication of endogenous lymphocytes might cause necrosis or apoptosis of tumor cells, which results in antigen presenting cells uptake of tumor antigens and the subsequent cross-presentation of these antigens to the adoptively transferred tumor-reactive CD8^+^ T cells [49].

The improved effectiveness of immunotherapy following a non-myeloablative lymphodepleting regimen encourages the researchers for more elegant conditioning regimens in selective or intensive ways. T_Reg_ cells might be selectively depleted with directed immunotoxins or suppressed by administering a cytokine such as tumor necrosis factor (TNF). Exogenous replenishment of certain cytokines may alleviate the sink effect coming from the endogenous T cells. Attempt of using cytokines such as IL-7, IL-15 and IL-21 might avoid suppressive function of IL-2 toward T_Reg_ cells. Intensive myeloablative preconditioning regimen with chemotherapy, total body irradiation and human stem cell transplantation (HSCT) is also under planned in human AIT protocol.

## Conclusions and perspectives

CAR-modified T cells constitute a very appealing approach to cancer immunotherapy. Despite the promising results obtained from numerous clinical trials following the infusion of CAR-modified T cells, some severe adverse reactions were reported [12,50,51]. Recent reports have highlighted some key issues and future directions to avoid these adverse events. Care selection of candidate target antigens is essential for improved efficacy and safety of the CAR-based therapy. Additional factors needed to be considered include the density of target molecules, accessibility of the epitope, affinity of the scFv, flexibility of hinge, property of the signaling, etc. New elements are being evaluated including a controllable suicide gene like caspase 9 or HSV-TK as a safety switch [52], and a chemokine receptor for T cells to migrate to tumor site. Furthermore, the best sources of the selective T cell populations are getting attention for CAR T cell therapy. Many factors may contribute to the improved quality of the cells such as closed cell processing system, new activation/expansion methods, selection of T cell subsets with therapeutic potentials, improved culture condition, etc. Current protocols for cell bioengineering need to be simplified to facilitate widespread application of such treatment strategy. Finally, because it is technically challenging, labor intensive and very costly, most of the studies have been conducted in academic centers. Future work needs collective effort from regulatory organizations, biological companies, medical centers and academic institutions. We believe these collaborations, some of which are already in progress, would lead to the development of the comprehensive approaches to maximize the efficacy of the cancer immunotherapy.

## Competing interests

The authors declare that they have no competing interests.

## Authors’ contributions

All authors have contributed to write and revise the manuscripts. All authors read and approved the final manuscript.

## References

[B1] Eshhar Z Waks T Gross G Schindler DG Specific activation and targeting of cytotoxic lymphocytes through chimeric single chains consisting of antibody-binding domains and the gamma or zeta subunits of the immunoglobulin and T-cell receptors Proc Natl Acad Sci USA 1993 90 2 720 724 10.1073/pnas.90.2.720 8421711 PMC45737

[B2] Cheadle EJ Sheard V Hombach AA Chmielewski M Riet T Berrevoets C Schooten E Lamers C Abken H Debets R Chimeric antigen receptors for T-cell based therapy Methods Mol Biol 2012 907 645 666 22907378 10.1007/978-1-61779-974-7_36

[B3] Kochenderfer JN Rosenberg SA Treating B-cell cancer with T cells expressing anti-CD19 chimeric antigen receptors Nat Rev Clin Oncol 2013 10 5 267 276 10.1038/nrclinonc.2013.46 23546520 PMC6322669

[B4] Porter DL Levine BL Kalos M Bagg A June CH Chimeric antigen receptor-modified T cells in chronic lymphoid leukemia N Engl J Med 2011 365 8 725 733 10.1056/NEJMoa1103849 21830940 PMC3387277

[B5] Brentjens RJ Davila ML Riviere I Park J Wang X Cowell LG Bartido S Stefanski J Taylor C Olszewska M CD19-Targeted T cells rapidly induce molecular remissions in adults with chemotherapy-refractory acute lymphoblastic leukemia Sci Transl Med 2013 5 177 177ra38 10.1126/scitranslmed.3005930 23515080 PMC3742551

[B6] Grupp SA Kalos M Barrett D Aplenc R Porter DL Rheingold SR Teachey DT Chew A Hauck B Wright JF Chimeric antigen receptor-modified T cells for acute lymphoid leukemia N Engl J Med 2013 368 16 1509 1518 10.1056/NEJMoa1215134 23527958 PMC4058440

[B7] Xu XJ Zhao HZ Tang YM Efficacy and safety of adoptive immunotherapy using anti-CD19 chimeric antigen receptor transduced T-cells: a systematic review of phase I clinical trials Leuk Lymphoma 2013 54 2 255 260 10.3109/10428194.2012.715350 22897728

[B8] Haso W Lee DW Shah NN Stetler-Stevenson M Yuan CM Pastan IH Dimitrov DS Morgan RA FitzGerald DJ Barrett DM Anti-CD22-chimeric antigen receptors targeting B-cell precursor acute lymphoblastic leukemia Blood 2013 121 7 1165 1174 10.1182/blood-2012-06-438002 23243285 PMC3575759

[B9] Park JR Digiusto DL Slovak M Wright C Naranjo A Wagner J Meechoovet HB Bautista C Chang WC Ostberg JR Adoptive transfer of chimeric antigen receptor re-directed cytolytic T lymphocyte clones in patients with neuroblastoma Mol Ther 2007 15 4 825 833 17299405 10.1038/sj.mt.6300104

[B10] Louis CU Savoldo B Dotti G Pule M Yvon E Myers GD Rossig C Russell HV Diouf O Liu E Antitumor activity and long-term fate of chimeric antigen receptor-positive T cells in patients with neuroblastoma Blood 2011 118 23 6050 6056 10.1182/blood-2011-05-354449 21984804 PMC3234664

[B11] Lamers CH Sleijfer S van Steenbergen S van Elzakker P van Krimpen B Groot C Vulto A den Bakker M Oosterwijk E Debets R Treatment of metastatic renal cell carcinoma with CAIX CAR-engineered T cells: clinical evaluation and management of on-target toxicity Mol Ther 2013 21 4 904 912 10.1038/mt.2013.17 23423337 PMC5189272

[B12] Morgan RA Yang JC Kitano M Dudley ME Laurencot CM Rosenberg SA Case report of a serious adverse event following the administration of T cells transduced with a chimeric antigen receptor recognizing ERBB2 Mol Ther 2010 18 4 843 851 10.1038/mt.2010.24 20179677 PMC2862534

[B13] Kandalaft LE Powell DJ Jr Coukos G A phase I clinical trial of adoptive transfer of folate receptor-alpha redirected autologous T cells for recurrent ovarian cancer J Transl Med 2012 10 157 10.1186/1479-5876-10-157 22863016 PMC3439340

[B14] Schlimper C Hombach AA Abken H Schmidt-Wolf IG Improved activation toward primary colorectal cancer cells by antigen-specific targeting autologous cytokine-induced killer cells Clin Dev Immunol 2012 201 2 238924 22481963 10.1155/2012/238924 PMC3310246

[B15] Morgan RA Johnson LA Davis JL Zheng Z Woolard KD Reap EA Feldman SA Chinnasamy N Kuan CT Song H Recognition of glioma stem cells by genetically modified T cells targeting EGFRvIII and development of adoptive cell therapy for glioma Hum Gene Ther 2012 23 10 1043 1053 10.1089/hum.2012.041 22780919 PMC3472555

[B16] Kloss CC Condomines M Cartellieri M Bachmann M Sadelain M Combinatorial antigen recognition with balanced signaling promotes selective tumor eradication by engineered T cells Nat Biotechnol 2013 31 1 71 75 23242161 10.1038/nbt.2459 PMC5505184

[B17] Shirasu N Kuroki M Functional design of chimeric T-cell antigen receptors for adoptive immunotherapy of cancer: architecture and outcomes Anticancer Res 2012 32 6 2377 2383 22641678

[B18] Hudecek M Lupo-Stanghellini MT Kosasih PL Sommermeyer D Jensen MC Rader C Riddell SR Receptor affinity and extracellular domain modifications affect tumor recognition by ROR1-specific chimeric antigen receptor T cells Clin Cancer Res 2013 19 12 3153 3164 10.1158/1078-0432.CCR-13-0330 23620405 PMC3804130

[B19] Chinnasamy D Tran E Yu Z Morgan RA Restifo NP Rosenberg SA Simultaneous targeting of tumor antigens and the tumor vasculature using T lymphocyte transfer synergize to induce regression of established tumors in mice Cancer Res 2013 73 11 3371 3380 10.1158/0008-5472.CAN-12-3913 23633494 PMC3686092

[B20] Zhang T Barber A Sentman CL Chimeric NKG2D modified T cells inhibit systemic T-cell lymphoma growth in a manner involving multiple cytokines and cytotoxic pathways Cancer Res 2007 67 22 11029 11036 10.1158/0008-5472.CAN-07-2251 18006849

[B21] Hombach A Hombach AA Abken H Adoptive immunotherapy with genetically engineered T cells: modification of the IgG1 Fc spacer domain in the extracellular moiety of chimeric antigen receptors avoids off-target activation and unintended initiation of an innate immune response Gene Ther 2010 17 10 1206 1213 10.1038/gt.2010.91 20555360

[B22] Guest RD Hawkins RE Kirillova N Cheadle EJ Arnold J O’Neill A Irlam J Chester KA Kemshead JT Shaw DM The role of extracellular spacer regions in the optimal design of chimeric immune receptors: evaluation of four different scFvs and antigens J Immunother 2005 28 3 203 211 10.1097/01.cji.0000161397.96582.59 15838376

[B23] Bridgeman JS Hawkins RE Bagley S Blaylock M Holland M Gilham DE The optimal antigen response of chimeric antigen receptors harboring the CD3zeta transmembrane domain is dependent upon incorporation of the receptor into the endogenous TCR/CD3 complex J Immunol 2010 184 12 6938 6949 10.4049/jimmunol.0901766 20483753

[B24] Savoldo B Ramos CA Liu E Mims MP Keating MJ Carrum G Kamble RT Bollard CM Gee AP Mei Z CD28 Costimulation improves expansion and persistence of chimeric antigen receptor-modified T cells in lymphoma patients J Clin Invest 2011 121 5 1822 1826 10.1172/JCI46110 21540550 PMC3083795

[B25] Tammana S Huang X Wong M Milone MC Ma L Levine BL June CH Wagner JE Blazar BR Zhou X 4-1BB And CD28 signaling plays a synergistic role in redirecting umbilical cord blood T cells against B-cell malignancies Hum Gene Ther 2010 21 1 75 86 10.1089/hum.2009.122 19719389 PMC2861957

[B26] Finney HM Akbar AN Lawson AD Activation of resting human primary T cells with chimeric receptors: costimulation from CD28, inducible costimulator, CD134, and CD137 in series with signals from the TCR zeta chain J Immunol 2004 172 1 104 113 14688315 10.4049/jimmunol.172.1.104

[B27] Milone MC Fish JD Carpenito C Carroll RG Binder GK Teachey D Samanta M Lakhal M Gloss B Danet-Desnoyers G Chimeric receptors containing CD137 signal transduction domains mediate enhanced survival of T cells and increased antileukemic efficacy in vivo Mol Ther 2009 17 8 1453 1464 10.1038/mt.2009.83 19384291 PMC2805264

[B28] Shen CJ Yang YX Han EQ Cao N Wang YF Wang Y Zhao YY Zhao LM Cui J Gupta P Chimeric antigen receptor containing ICOS signaling domain mediates specific and efficient antitumor effect of T cells against EGFRvIII expressing glioma J Hematol Oncol 2013 6 33 10.1186/1756-8722-6-33 23656794 PMC3658918

[B29] Klebanoff CA Gattinoni L Restifo NP Sorting through subsets: which T-cell populations mediate highly effective adoptive immunotherapy? J Immunother 2012 35 9 651 660 10.1097/CJI.0b013e31827806e6 23090074 PMC3501135

[B30] Berger C Jensen MC Lansdorp PM Gough M Elliott C Riddell SR Adoptive transfer of effector CD8+ T cells derived from central memory cells establishes persistent T cell memory in primates J Clin Invest 2008 118 1 294 305 10.1172/JCI32103 18060041 PMC2104476

[B31] Gattinoni L Lugli E Ji Y Pos Z Paulos CM Quigley MF Almeida JR Gostick E Yu Z Carpenito C A human memory T cell subset with stem cell-like properties Nat Med 2011 17 10 1290 1297 10.1038/nm.2446 21926977 PMC3192229

[B32] Wang X Naranjo A Brown CE Bautista C Wong CW Chang WC Aguilar B Ostberg JR Riddell SR Forman SJ Phenotypic and functional attributes of lentivirus-modified CD19-specific human CD8+ central memory T cells manufactured at clinical scale J Immunother 2012 35 9 689 701 10.1097/CJI.0b013e318270dec7 23090078 PMC3525345

[B33] Turtle CJ Riddell SR Artificial antigen-presenting cells for use in adoptive immunotherapy Cancer J 2010 16 4 374 381 10.1097/PPO.0b013e3181eb33a6 20693850 PMC2929753

[B34] Antony PA Paulos CM Ahmadzadeh M Akpinarli A Palmer DC Sato N Kaiser A Hinrichs CS Klebanoff CA Tagaya Y Interleukin-2-dependent mechanisms of tolerance and immunity in vivo J Immunol 2006 176 9 5255 5266 16621991 10.4049/jimmunol.176.9.5255 PMC1473163

[B35] Mueller YM Makar V Bojczuk PM Witek J Katsikis PD IL-15 enhances the function and inhibits CD95/Fas-induced apoptosis of human CD4+ and CD8+ effector-memory T cells Int Immunol 2003 15 1 49 58 10.1093/intimm/dxg013 12502725

[B36] Rao RR Li Q Odunsi K Shrikant PA The mTOR kinase determines effector versus memory CD8+ T cell fate by regulating the expression of transcription factors T-bet and eomesodermin Immunity 2010 32 1 67 78 10.1016/j.immuni.2009.10.010 20060330 PMC5836496

[B37] Colovos C Villena-Vargas J Adusumilli PS Safety and stability of retrovirally transduced chimeric antigen receptor T cells Immunotherapy 2012 4 9 899 902 10.2217/imt.12.91 23046233

[B38] Scholler J Brady TL Binder-Scholl G Hwang WT Plesa G Hege KM Vogel AN Kalos M Riley JL Deeks SG Decade-long safety and function of retroviral-modified chimeric antigen receptor T cells Sci Transl Med 2012 4 132 132ra53 10.1126/scitranslmed.3003761 22553251 PMC4368443

[B39] McGarrity GJ Hoyah G Winemiller A Andre K Stein D Blick G Greenberg RN Kinder C Zolopa A Binder-Scholl G Patient monitoring and follow-up in lentiviral clinical trials J Gene Med 2013 15 2 78 82 10.1002/jgm.2691 23322669

[B40] Schroers R Hildebrandt Y Hasenkamp J Glass B Lieber A Wulf G Piesche M Gene transfer into human T lymphocytes and natural killer cells by Ad5/F35 chimeric adenoviral vectors Exp Hematol 2004 32 6 536 546 10.1016/j.exphem.2004.03.010 15183894

[B41] Maiti SN Huls H Singh H Dawson M Figliola M Olivares S Rao P Zhao YJ Multani A Yang G Sleeping beauty system to redirect T-cell specificity for human applications J Immunother 2013 36 2 112 123 10.1097/CJI.0b013e3182811ce9 23377665 PMC3568214

[B42] Nakazawa Y Huye LE Salsman VS Leen AM Ahmed N Rollins L Dotti G Gottschalk SM Wilson MH Rooney CM PiggyBac-mediated cancer immunotherapy using EBV-specific cytotoxic T-cells expressing HER2-specific chimeric antigen receptor Mol Ther 2011 19 12 2133 2143 10.1038/mt.2011.131 21772253 PMC3242651

[B43] Barrett DM Zhao Y Liu X Jiang S Carpenito C Kalos M Carroll RG June CH Grupp SA Treatment of advanced leukemia in mice with mRNA engineered T cells Hum Gene Ther 2011 22 12 1575 1586 10.1089/hum.2011.070 21838572 PMC3237694

[B44] Dudley ME Yang JC Sherry R Hughes MS Royal R Kammula U Robbins PF Huang J Citrin DE Leitman SF Adoptive cell therapy for patients with metastatic melanoma: evaluation of intensive myeloablative chemoradiation preparative regimens J Clin Oncol 2008 26 32 5233 5239 10.1200/JCO.2008.16.5449 18809613 PMC2652090

[B45] Rosenberg SA Yang JC Sherry RM Kammula US Hughes MS Phan GQ Citrin DE Restifo NP Robbins PF Wunderlich JR Durable complete responses in heavily pretreted patients with metastatic melanoma using T cell transfer immunotherapy Clin Cancer Res 2011 17 13 4550 4557 10.1158/1078-0432.CCR-11-0116 21498393 PMC3131487

[B46] Kalos M Levine BL Porter DL Katz S Grupp SA Bagg A June CH T cells with chimeric antigen receptors have potent antitumor effects and can establish memory in patients with advanced leukemia Sci Transl Med 2011 3 95 95ra73 10.1126/scitranslmed.3002842 21832238 PMC3393096

[B47] Curiel TJ Coukos G Zou L Alvarez X Cheng P Mottram P Evdemon-Hogan M Conejo-Garcia JR Zhang L Burow M Specific recruitment of regulatory T cells in ovarian carcinoma fosters immune privilege and predicts reduced survival Nature Med 2004 10 9 942 949 10.1038/nm1093 15322536

[B48] Gattinoni L Finkelstein SE Klebanoff CA Antony PA Palmer DC Spiess PJ Hwang LN Yu Z Wrzesinski C Heimann DM Removal of homeostatic cytokine sinks by lymphodepletion enhances the efficacy of adoptively transferred tumour-specific CD8+ T cells J Exp Med 2005 202 7 907 912 10.1084/jem.20050732 16203864 PMC1397916

[B49] Russo V Tanzarella S Dalerba P Rigatti D Rovere P Villa A Bordignon C Traversari C Dendritic cells acquire the MAGE-3 human tumour antigen from apoptotic cells and induce a class I-restricted T cell response Proc Natl Acad Sci USA 2000 97 5 2185 2190 10.1073/pnas.040540197 10681453 PMC15775

[B50] Brentjens R Yeh R Bernal Y Riviere I Sadelain M Treatment of chronic lymphocytic leukemia with genetically targeted autologous T cells: case report of an unforeseen adverse event in a phase I clinical trial Mol Ther 2010 18 4 666 668 10.1038/mt.2010.31 20357779 PMC2862525

[B51] Kochenderfer JN Dudley ME Feldman SA Wilson WH Spaner DE Maric I Stetler-Stevenson M Phan GQ Hughes MS Sherry RM B-cell depletion and remissions of malignancy along with cytokine-associated toxicity in a clinical trial of anti-CD19 chimeric- antigen -receptor-transduced T cells Blood 2012 119 12 2709 2720 10.1182/blood-2011-10-384388 22160384 PMC3327450

[B52] Di Stasi A Tey SK Dotti G Fujita Y Kennedy-Nasser A Martinez C Straathof K Liu E Durett AG Grilley B Inducible apoptosis as a safety switch for adoptive cell therapy N Engl J Med 2011 365 18 1673 1683 10.1056/NEJMoa1106152 22047558 PMC3236370

